# Assessment of Pharmacokinetics and Metabolism Profiles of SCH 58261 in Rats Using Liquid Chromatography–Mass Spectrometric Method

**DOI:** 10.3390/molecules25092209

**Published:** 2020-05-08

**Authors:** Yuri Park, Min-Ho Park, Jin-Ju Byeon, Seok-Ho Shin, Byeong ill Lee, Jang-mi Choi, Nahye Kim, Seo-jin Park, Min-jae Park, Jeong-hyeon Lim, Young G. Shin

**Affiliations:** College of Pharmacy and Institute of Drug Research and Development, Chungnam National University, 99, Daehak-ro, Yuseong-gu, Daejeon 34134, Korea; yuri.park.cnu@gmail.com (Y.P.); minho.park.cnu@gmail.com (M.-H.P.); jinju.byeon.cnu@gmail.com (J.-J.B.); seokho.shin.cnu@gmail.com (S.-H.S.); byungill.lee.cnu@gmail.com (B.i.L.); jangmi.choi.cnu@gmail.com (J.-m.C.); nahye.kim.cnu@gmail.com (N.K.); seojin.park.cnu@gmail.com (S.-j.P.); minjae.park.cnu@gmail.com (M.-j.P.); jeonghyeon.Lim.cnu@gmail.com (J.-h.L.)

**Keywords:** SCH 58261, A_2A_ receptor inhibitor, LC–MS/MS, pharmacokinetics, metabolism, bioavailability

## Abstract

5-Amino-7-(2-phenylethyl)-2-(2-furyl)-pyrazolo(4,3-e)-1,2,4-triazolo(1,5-c) pyrimidine (SCH 58261) is one of the new chemical entities that has been developed as an adenosine A_2A_ receptor antagonist. Although SCH 58261 has been reported to be beneficial, there is little information about SCH 58261 from a drug metabolism or pharmacokinetics perspective. This study describes the metabolism and pharmacokinetic properties of SCH 58261 in order to understand its behaviors in vivo. Rats were used as the in vivo model species. First, an LC–MS/MS method was developed for the determination of SCH 58261 in rat plasma. A GastroPlus™ simulation, in vitro microsomal metabolic stability, and bile duct-cannulated studies were also performed to understand its pharmacokinetic profile. The parameter sensitivity analysis of GastroPlus™ was used to examine the factors that influence exposure when the drug is orally administered. The factors are as follows: permeability, systemic clearance, renal clearance, and liver first-pass effect. In vitro microsomal metabolic stability indicates how much the drug is metabolized. The extrapolated hepatic clearance value of SCH 58261 was 39.97 mL/min/kg, indicating that the drug is greatly affected by hepatic metabolism. In vitro microsomal metabolite identification studies revealed that metabolites produce oxidized and ketone-formed metabolites via metabolic enzymes in the liver. The bile duct-cannulated rat study, after oral administration of SCH 58261, showed that a significant amount of the drug was excreted in feces. These results imply that the drug is not absorbed well in the body after oral administration. Taken together, SCH 58261 showed quite a low bioavailability when administered orally and this was likely due to significantly limited absorption, as well as high metabolism in vivo.

## 1. Introduction

The blockade of the adenosine A_2A_ receptor in striatopallidal neurons is known to reduce the postsynaptic effect of dopamine depletion and the motor deficit of Parkinson’s disease (PD). Because of these properties, adenosine A_2A_ receptor antagonists are well known as good target drug candidates for the treatment of PD [1,2,3,4,5,6,7].

Recently, as interest in immunotherapy has increased, many articles and papers have shown that adenosine pathways are also involved in immunosuppression [8,9,10]. The pathway of adenosine production by 5′-nucleotidase is one of the immunosuppressive pathways [8,9]. The expression of 5′-nucleotidase enhances tumor progression and metastasis [8,9,11,12]. CD39/CD73 expressed in tumors convert Adenosine triphosphate into adenosine [8,9,10,12,13,14]. This adenosine binds to the adenosine A_2A_ receptor to increase cAMP, and this cAMP inhibits immune response factors such as natural killer cells, dendritic cells, and T cells [10,11,13,15]. This chain reaction may put the brakes on the anti-tumor immune response against the tumor, while it enhances tumor progression and metastasis [10,11,13,14]. Conversely, when the adenosine A_2A_ receptor antagonist is applied to inhibit the receptor, an immune response may occur, thus exhibiting an anti-tumor effect [8,10,11,13,14,15].

**Figure 1:**
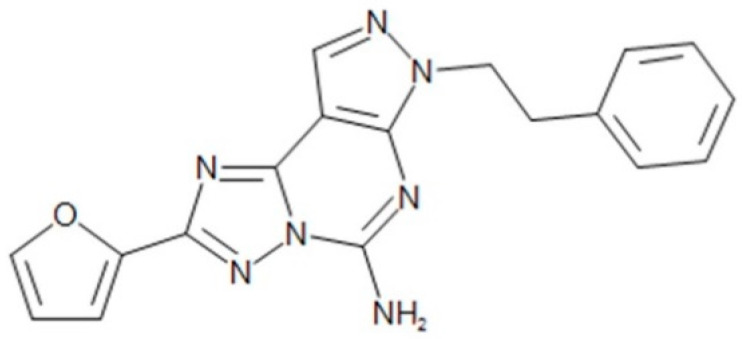
Structure of 5-amino-7-(2-phenylethyl)-2-(2-furyl)-pyrazolo(4,3-e)-1,2,4-triazolo(1,5-c) pyrimidine (SCH 58261).

5-Amino-7-(2-phenylethyl)-2-(2-furyl)-pyrazolo(4,3-e)-1,2,4-triazolo(1,5-c) pyrimidine (SCH 58261) is classified as an adenosine A_2A_ receptor antagonist [6,7,16,17,18,19] (Figure 1). The non-xanthine heterocyclic compound SCH 58261 is a new, potent and selective A_2A_ adenosine receptor antagonist [18]. This drug particularly binds to the A_2A_ receptor, among the various adenosine receptor subtypes [18,19]. It is known that SCH 58261 is potent against cAMP production as well as binding [17,18,20]. SCH 58261 is one of the new chemical entities that has been developed as an adenosine A_2A_ receptor antagonist. Since then, SCH 58261 has been widely used as a reference drug in the development of adenosine A_2A_ receptor antagonists in many articles [21,22,23,24,25]. However, although SCH 58261 has been reported to be beneficial, there is little information about SCH 58261 in terms of its absorption, distribution, metabolism, and excretion (ADME) as well as its pharmacokinetics (PK) perspectives in animals and humans. 

The response of a drug in in vivo animal models conventionally depends on its absorption, distribution, metabolism, and elimination associated with the pharmacokinetic factors [26]. Therefore, the ADME knowledge in the chemical series of drugs is essential for successful structural optimization and design during lead optimization and the candidate selection process. Bioavailability quantifies the proportion of a drug that is absorbed and available to produce systemic effects [27,28]. Oral bioavailability is one of the key factors frequently assessed in drug development. In general, the factors affecting drug bioavailability after oral administration include (but are not limited to) absorption and metabolism [26].

The purpose of this study was to explore the metabolism and pharmacokinetic properties of SCH 58261 to understand its behaviors in vivo. Male Sprague–Dawley (SD) rats (8 weeks old) were used as an in vivo model species. First, an LC–MS/MS method was developed for the determination of SCH 58261 in rat plasma. GastroPlus™ is a tool to simulate the PK properties of drugs in virtual humans and animals [29]. Parameter sensitivity analysis is one of the built-in modules of GastroPlus™, which is useful for understanding the sensitivities in PK profiles between various PK parameters [30,31,32]. In vitro microsomal metabolic stability, in vitro metabolite identification, and bile duct-cannulated studies were also conducted to understand the PK characteristics of SCH 58261. In vitro microsomal metabolic stability can be determined by in vitro half-life (t_1/2_) and intrinsic clearance (CL_int_) using a simple in vitro experiment [33,34,35]. This value can be used to extrapolate in vivo intrinsic clearance and hepatic clearance [33,35]. Since the liver is considered to be one of the main organs involved in drug metabolism, the hepatic clearance value obtained from the in vitro study would make it possible to predict how much metabolism could occur in the liver [33,35]. The identification of metabolites using rat liver microsomes is an experiment that can easily predict metabolites generated by the liver metabolic enzymes [36]. The bile duct-cannulated rat study is a test to see the amount of drug excreted in each sample after collecting the feces, urine, and bile, which is obtained by connecting the tube to the bile duct of the rat [37]. Overall, the following factors such as permeability, systemic clearance, renal clearance, and liver first-pass effect, etc., will be explored from various in vitro and in vivo experiments in this study to understand the drug metabolism and pharmacokinetic properties of SCH 58261.

## 2. Results and Discussions

### 2.1. Development of SCH 58261 Quantification Method

#### 2.1.1. Calibration Curve, Accuracy and Precision

**Figure 2:**
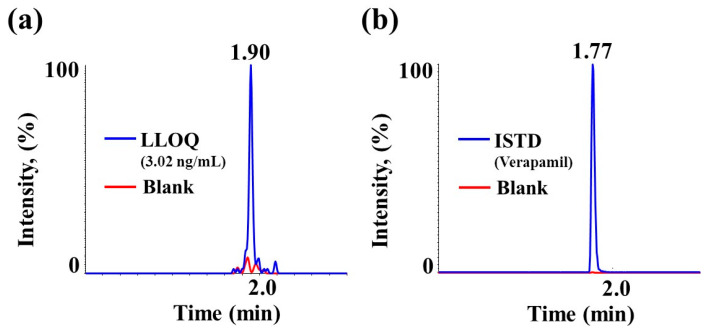
Representative chromatogram of (**a**) lower limits of quantification (LLOQ) (3.02 ng/mL) for SCH 58261 and (**b**) internal standard (ISTD) (verapamil) with blank matrix in rat plasma.

Representative chromatograms of the lower limits of quantification (LLOQ, 3.02 ng/mL) and the internal standard (ISTD) are shown in Figure 2. The signal-to-noise of LLOQ was more than 10 and has sensitivity. This sensitivity is sufficient to cover all time courses for this rat PK study with a dose level of 1 mg/kg of SCH 58261.

**Figure 3:**
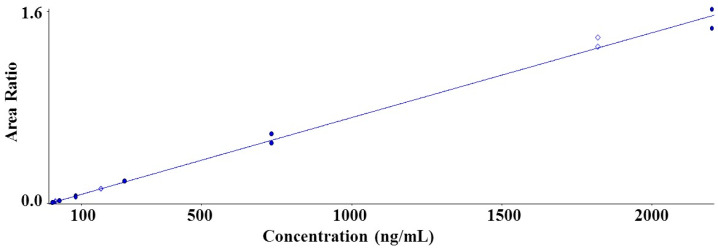
Representative calibration curve for SCH 58261 in rat plasma (r = 0.99414, range = 3.02~2200 ng/mL).

The calibration curve range was 3.02~2200 ng/mL. The run contained duplicate calibration curve standards (STD) at seven concentrations, quality control (QC) samples at three concentrations, and two blank samples. All calibration curves were well established using a weighted quadratic regression: 1/concentration^2^ (Figure 3). The correlation coefficient for evaluating the linearity of the calibration curve was ≥0.99 for SCH 58261.

In this method, the intra-/inter-day accuracy and precision were also determined by the triplicate analyses of QC samples at three concentrations and the results are shown in Table 1. The intra-/inter-day accuracy and precision values were all within ±25%. The results were sufficient enough to be used as a bioanalytical method for the quantification of SCH 58261 in rat plasma.

#### 2.1.2. Stability

The stability evaluation was conducted in four different environments: stock stability, short-term stability, long-term stability, and freeze–thaw stability. The stability results are shown in Table 2. All stability evaluations were proceeded by triplicates of QC samples and if the accuracy and precision (% CV) of QC samples were within ±25%, the results would be considered to be acceptable for the early discovery fit-for-purpose research. Since the accuracy and precision of four stability assessments were within ±25%, SCH 58261 in stock solution was determined to be stable for 28 days at −20 °C and SCH 58261 in rat plasma was determined to be stable for 4 hours at room temperature and 28 days at −80 °C. Also, SCH 58261 in rat plasma was also determined to be stable by three freeze–thaw cycles at −80 °C.

#### 2.1.3. Dilution Integrity

Dilution integrity was assessed with the dilution of SCH 58261 in rat plasma. This assessment was performed in triplicates of diluted QC (QC dil) by diluting five times with blank rat plasma and the results are shown in Table 3. As a result, accuracy and precision were within ±25% and a fivefold dilution of SCH 58261 with blank rat plasma was also satisfactory.

#### 2.1.4. Species-Dependent Matrix Effect

The species-dependent matrix effect assessed the matrix effect of other species (mouse, dog and human) against rat plasma. This assessment was performed on a rat plasma calibration curve with the inclusion of QC samples (high, medium, and low) using mouse, dog, and human plasma in triplicate to evaluate accuracy and precision. If there were no or little species-dependent matrix effects between species, the rat plasma calibration curve would be able to quantitate SCH 58261 in other species as well. Table 4 shows that the accuracy and precision of the QC samples were within ±25%, indicating that there was no significant species-dependent matrix effect between rat plasma and other species.

### 2.2. Application for A Pharmacokinetic Study in Rats

**Figure 4:**
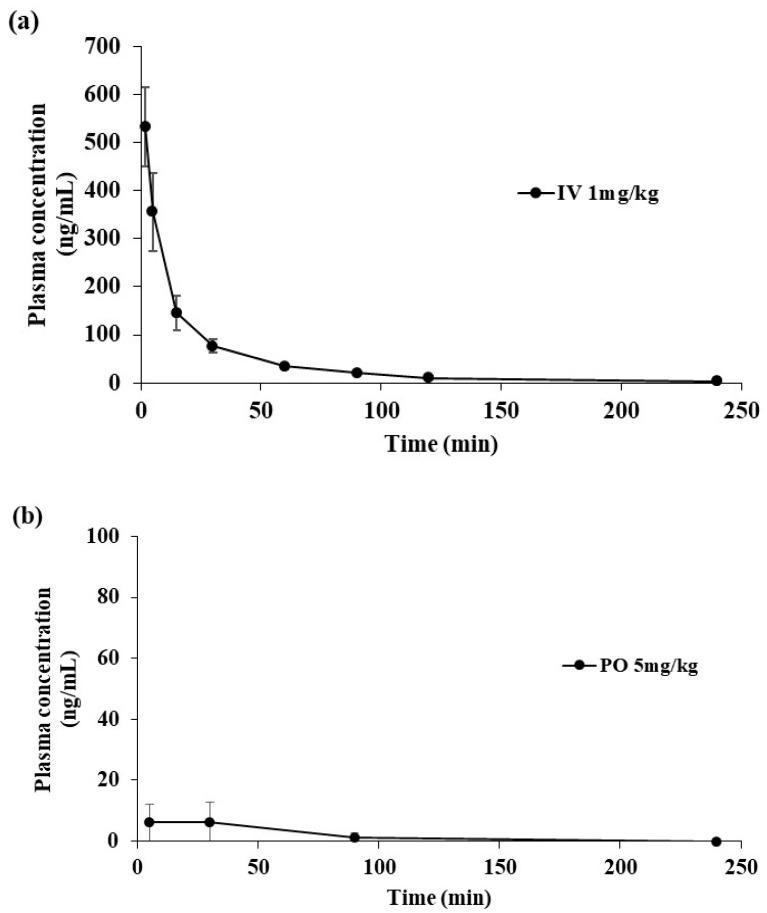
Concentration-time profiles of SCH 58261 in rat following intravenous (IV) administration at 1 mg/kg (**a**) and oral (PO) administration at 5 mg/kg (**b**).

This developed method was applied to determine the plasma concentration of SCH 58261 following intravenous administration at 1 mg/kg and oral administration at 5 mg/kg. The mean plasma concentration-time profiles of SCH 58261 are shown in Figure 4 and their estimated pharmacokinetic parameters are presented in Table 5. When administered intravenously, SCH 58261 was detectable up to 240 min. The assay was sensitive enough for the determination of SCH 58261 in rat plasma after oral and intravenous administration. The pharmacokinetic parameters were calculated using non-compartmental analysis (WinNonlin^®^). The maximum concentration (C_max_) of SCH 58261 after intravenous administration was 1135.44 ng/mL and the area under the curve (AUC_last_) was 11,528.45 min∙ng/mL. The clearance (CL) was 87.91 mL/min/kg. It was supposed that SCH 58261 was labile in vivo metabolic conditions based on the in vivo CL value. CL in vivo was greater than CL_H_ obtained from an in vitro metabolic stability study using rat liver microsomes. Therefore, it was considered that SCH 58261 had other elimination mechanisms in addition to the metabolism by the liver. The volume of distribution at steady state (V_ss_) was 3196.92 mL/kg. After oral administration, SCH 58261 was almost not detected in rat plasma. The maximum concentration (C_max_) of SCH 58261 after oral administration was 6.12 ng/mL and the area under the curve (AUC_last_) was only 15.29 min∙ng/mL. As a result, bioavailability (BA) was extremely low with a value of 0.03% in rats. Several factors contribute to this low BA, including low solubility, low absorption due to poor permeability, and a large first-pass effect. To investigate the root-cause of this low BA, a parameter sensitivity analysis (PSA) using GastroPlus™, an in vitro metabolic study, and bile duct-cannulated rat studies were conducted.

### 2.3. Parameter Sensitivity Analysis

**Figure 5:**
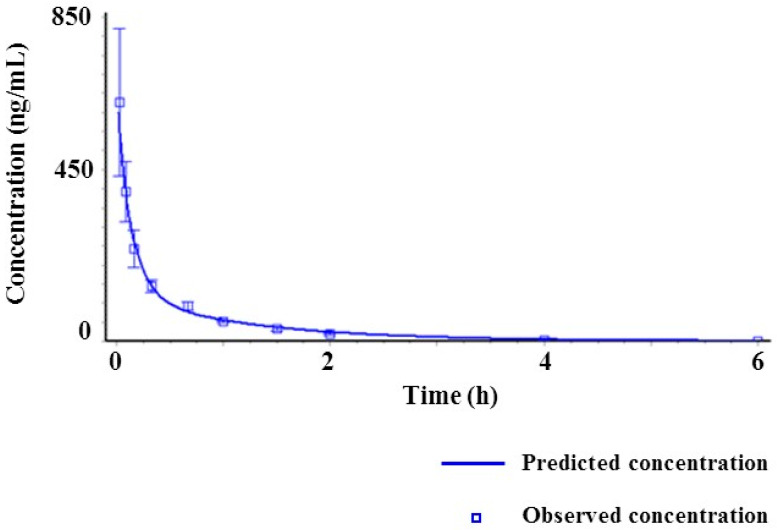
The simulated two-compartment model of SCH 58261 after IV administration using GastroPlus^™^, the predicted and observed time concentration profiles.

The in silico tool was used to determine which factors were significantly involved in poor drug exposure after oral administration of SCH 58261. In this study, GastroPlus™ was used to predict the PK characteristics of SCH 58261 [29]. GastroPlus™ is a tool to simulate the PK of drugs in virtual humans and animals based on in vitro and in vivo ADME/PK parameters. Parameter sensitivity analysis (PSA) is one of the built-in modules of GastroPlus™ and is also very useful to explore which PK parameters would be significant contributors to drug exposure in vivo after oral administration [30,31,32]. The following figure shows a typical simulated two-compartment model PK profile of SCH 58261 after IV administration (Figure 5).

**Figure 6:**
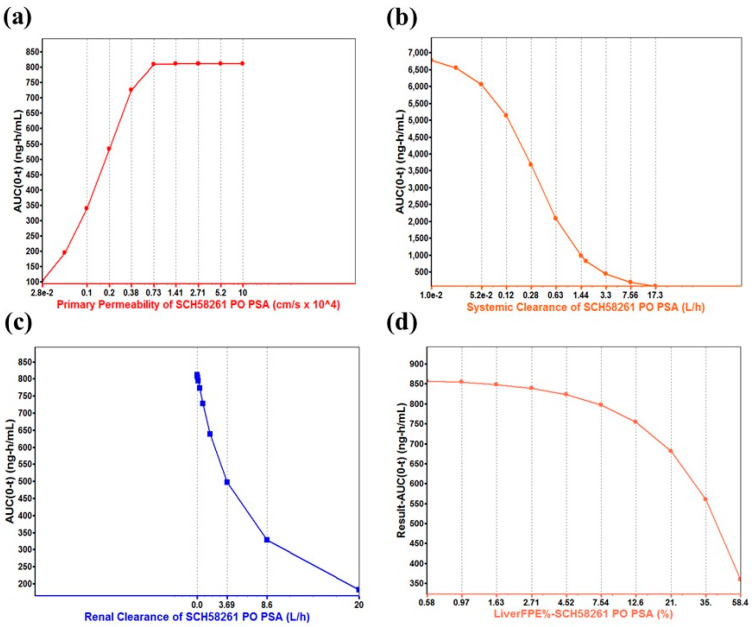
The parameter sensitivity analysis (PSA) of four factors that have significant effects on SCH 58261 AUC_last_ after oral administration in rats. (**a**) Permeability of SCH 58261, (**b**) systemic clearance of SCH 58261, (**c**) renal clearance of SCH 58261, and (**d**) liver first-pass effect (FPE)% of SCH 58261.

The PSA was conducted to examine the factors that significantly affect the drug exposure after oral administration of SCH 58261 in rats. A total of seven ADME/PK parameters were used to simulate the drug exposure: permeability, solubility, systemic clearance, renal clearance, liver first-pass effect (FPE), blood–plasma ratio, and unbound fraction. Eventually, these ADME/PK parameters were combined and further categorized into four PSA factors, including permeability, systemic clearance, renal clearance, and liver FPE%. Figure 6 shows the PSA results of SCH 58261.

### 2.4. Microsomal Metabolic Stability

The metabolic stability of SCH 58261 was determined based on the disappearance of the drug during incubation with rat liver microsomes. The in vitro t_1/2_ and intrinsic clearance (CL_int, in vitro_) were used as the parameters for assessment. Hepatic clearance (CL_H_) was calculated using these in vitro values. If SCH 58261 was rapidly metabolized, in vivo BA would probably be low.

The t_1/2_ was determined by plotting the remaining amount (%, the ratio of sample peak area/ISTD peak area) vs. time, then measuring the slope of the log-linear regression analysis [38]:

(1) $$t_{1/2}=0.693/\mathrm{slope}$$

The in vitro half-life was used to determine the CL_int, in vitro_, which is shown in the equation below [38,39].

(2) $${\mathrm{Cl}}_{\mathrm{int},\;\mathrm{in}\;\mathrm{vitro}}=\left(\frac{0.693}{t_{1/2}}\;\right)\times \left(\frac{\mathrm{microsome}\;\mathrm{volume}\;\left(\mathrm{mL}\right)}{\mathrm{amount}\;\mathrm{microsome}\;\mathrm{in}\;\mathrm{incubation}\left(\mathrm{mg}\right)}\right)$$

In vitro CL_int_ can be extrapolated to in vivo intrinsic clearance (CL_int, in vivo_) [38,39].

(3) $${\mathrm{Cl}}_{\mathrm{int}}={\mathrm{Cl}}_{\mathrm{int},\;\mathrm{in}\;\mathrm{vitro}}\times \left(\frac{44.8\;\mathrm{mg}\;\mathrm{microsomal}\;\mathrm{protein}}{g\;\mathrm{liver}}\right)\times \left(\frac{44\;g\;\mathrm{liver}}{\mathrm{kg}\;\mathrm{body}}\right)$$

Where the value of hepatic microsomal protein concentrations was 44.8 mg microsomal protein/g liver and the value of liver concentrations was 44 g liver/kg body weight in rats [39].

In the “well-stirred” model, the liver is represented by a single compartment, where the unbound concentration of a drug leaving the liver is in equilibrium with the intracellular unbound concentration in the hepatocytes [40]. The CL_H_ in the “well-stirred” model is expressed as shown in the formula below [40].

(4) $${\mathrm{Cl}}_{H}=\left(\frac{Q\times {\mathrm{Cl}}_{\mathrm{int}}}{Q+{\mathrm{Cl}}_{\mathrm{int}}}\right)$$

Where the value of Q, hepatic blood flow, was 55.8 mL/min/kg in rats [39].

The results from the microsomal metabolic stability experiment showed that 1.5 µg/mL of SCH 58261 disappeared almost within 60 min. The t_1/2_ and clearance obtained from in vitro microsomal metabolic stability experiments are shown in Table 6.

The half-life of the SCH 58261 in rat liver microsomes was short (10.95 min) and the in vitro intrinsic clearance was 0.063 mL/min/mg. The scaled in vivo intrinsic clearance value of SCH 58261 was 124.80 mL/min/kg and the extrapolated hepatic clearance value of SCH 58261 was 39.97 mL/min/kg in the rat. Assuming a rat liver blood flow of 55 mL/min/kg, the hepatic clearance value of SCH 58261 appears to have a high clearance (~72% of the rat liver blood flow). Therefore, one of the possible reasons that SCH 58261 showed low drug exposure after the oral administration would be possible due to the high metabolism of rat liver in vivo.

### 2.5. In Vitro Metabolite Identification

**Figure 7:**
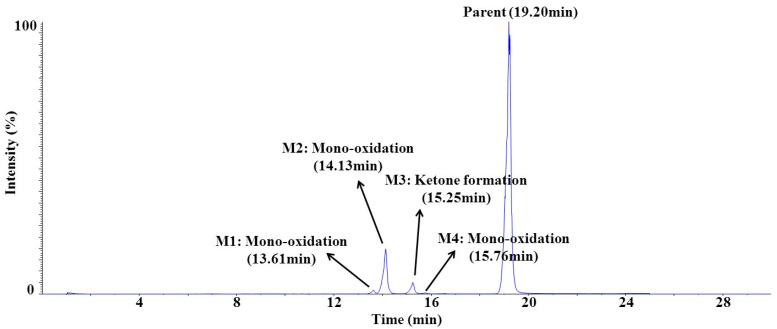
Chromatographic separation of in vitro rat liver microsomes.

**Figure 8:**
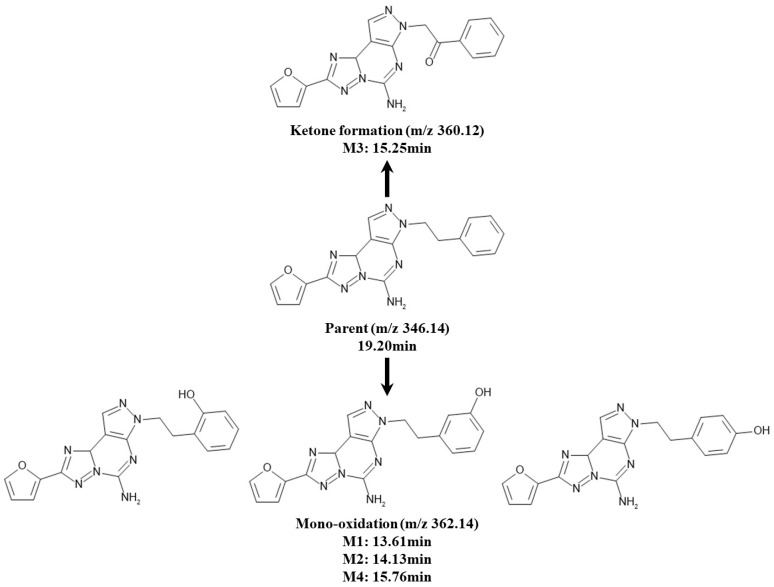
Metabolic pathways and proposed metabolite structures of SCH 58261 in vitro.

Under the described experimental conditions, four chromatographic peaks were observed from the in vitro metabolite identification study samples incubated in rat liver microsomes fortified with uridine 5′-diphosphoglucuronic acid triammonium salt (UDPGA) and glutathione (GSH). The fragmentation pattern and the retention time of SCH 58261 were used as a reference to compare those of the metabolites during the metabolite identification. The chromatographic separation of SCH 58261 and its metabolites are shown in Figure 7. All four chromatographic peaks were smaller than the parent SCH 58261 peak. For each peak, two types of metabolism were considered from the fragmentation patterns through MS/MS results: mono-oxidation and ketone formation (Table 7). The metabolic pathway of SCH 58261 is described in Figure 8. M1, M2, and M4 were mono-oxidized metabolites, and M3 was a metabolite-formed ketone. The retention times of M1, M2, M3, and M4 were 13.61, 14.13, 15.25, and 15.76 min, respectively. The ionization efficiency of SCH 58261 and its metabolites might be different and therefore this data would not be sufficient to provide quantitative information about SCH 58261 and its metabolites; however, this metabolite profile and identification data would be able to provide at least some insights into how SCH 58261 would be metabolized in vivo.

### 2.6. Bile Duct-Cannulated Rat Study

After oral administration of SCH 58261, the recovery of the drug in the bile, urine, and feces is summarized in Table 8. Over the 24 h collection period, 28.38% of SCH 58261 was recovered. SCH 58261 was excreted 0.02% in the urine, 0.19% in the bile, and 28.17% in the feces. This result implies that a significant amount of SCH 58261 was excreted through feces without gastrointestinal (GI) absorption. Some metabolites such as M1 and M2 were also observed in rat urine, but a relative amount of these metabolites based on the peak intensities compared to SCH58261 appeared to be minimal (data not shown). Since a large amount of SCH 58261 was excreted in the feces, the drug appeared to be poorly absorbed. The remaining drug would have been significantly metabolized.

Based on these results, the following conclusion can be possibly deduced. After oral administration of SCH 58261, approximately 28% of the administered drug was excreted into feces without absorption in 24 h. Even after the absorption of SCH 58261, at least 72% of the absorbed drug could be metabolized by the first-pass effect. Also, the drug could be metabolized by intestinal cytochrome P450 (CYPs) and other metabolic enzymes during the absorption process. As a result, the amount of SCH 58261 arriving at the systemic circulation would be quite low compared to the total administered drug amount.

## 3. Materials and Methods

### 3.1. Materials

SCH 58261 was purchased from MedChem Express (Monmouth Junction, NJ, USA). Uridine 5’-diphosphoglucuronic acid triammonium salt (UDPGA), nicotinamide adenine dinucleotide phosphate reduced (NADPH), glutathione (GSH) and verapamil, which was used for the internal standard (ISTD), were purchased from Sigma-Aldrich (St Louis, MO, USA). Male SD rat liver microsomes were purchased from Corning Incorporated (Corning, NY, USA). Acetonitrile (ACN) of HPLC grade was purchased from Honeywell Burdick & Jackson (Ulsan, Korea). Distilled water (DW) of HPLC grade was purchased from Samchun Chemical (Gyeonggi-do, Korea). All other chemicals and solvents were commercial products of analytical or reagent grade and were used without further purification.

### 3.2. Stock Solution

The stock solution of SCH 58261 was prepared by dissolving the drug in dimethyl sulfoxide (DMSO) at a concentration of 1 mg/mL and storing it in a refrigerator at −20 °C until use. The 0.1 mg/mL sub-stock solution was made by spiking 100 µL of stock solution in 900 µL of DMSO. ISTD was prepared by dissolving verapamil in ACN at a concentration of 100 ng/mL.

### 3.3. Preparation of Calibration Curve STD and QC Samples

The sub-stock solution of SCH 58261 was further diluted with DMSO to give a series of calibration curve standards, with concentrations ranging from 3.02 to 2200 ng/mL. The calibration curve standard samples were prepared in duplicate and the standard curves were obtained by establishing a quadratic regression function, with an equation y = ax^2^ + bx + c after 1/concentration^2^ weighting. QC samples with concentrations of 15.02 (QC low), 165.46 (QC medium) and 1820 (QC high) ng/mL were prepared in the same way as the calibration curve STDs.

### 3.4. Sample Preparation and Extraction Procedures

For each 20 µL of blank rat plasma, 4 µL of the STD or QC working solutions were added and vortexed for 1 min to mix. On the other hand, for blank samples, 4 µL of make-up solution (DMSO) was added to blank rat plasma. For study samples, 4 µL of make-up solution (DMSO) was also added to rat PK study samples to assure the same matrix conditions as STD and QC samples. Then, 100 µL of ACN containing 100 ng/mL verapamil as ISTD was added to the mixture for protein precipitation. After vortexing for 1 min and centrifuging at 10,000 rpm for 5 min, the supernatant was transferred to another Eppendorf tube. Finally, the samples were diluted three times with DW and the mixture was transferred to an LC vial for LC–MS/MS analysis.

### 3.5. Equipments and Chromatographic Conditions

The liquid chromatography–mass spectrometry system is composed of a Shimadzu CBM-20A HPLC pump controller (Shimadzu Corporation, Columbia, MD, USA), two Shimadzu LC-20AD pumps, a CTC HTS PAL auto-sampler (LEAP Technologies, Carrboro, NC, USA) and a Triple time-of-flight (TripleTOF^®^) 5600 mass spectrometer (Sciex, Foster City, CA, USA) with an electrospray ionization (ESI) source. The chromatographic separation was achieved on a Phenomenex Kinetex XB-C18 column (2.1 × 100 mm for metabolite identification and 2.1 × 50 mm for method qualification), and the column temperature was set to 50 °C. The mobile phase consisted of (A) 0.1% formic acid in water and (B) 0.1% formic acid in acetonitrile, with a binary flow rate of 0.4 mL/min and an injection volume of 10 μL. The LC gradients for SCH 58261 quantification and metabolite identification are summarized in Table 9.

The TripleTOF^®^ 5600 mass spectrometer was equipped with a TurboIonSpray^®^ ion source Electrospray ionization (Sciex, Redwood City, CA, USA) was used for sample introduction and ionization in the positive ion mode. High-purity nitrogen gas was used for the nebulizer/Duospray^™^ and curtain gases. Source-dependent parameters optimized were as follows: GS1 and GS2—both 50 psi; ion spray voltage—5500 V; temperature—500 °C, with a curtain gas flow of 30 L/min. The compound-dependent parameters such as the declustering potential (DP) and collision energy (CE) were optimized during tuning as 160 V and 35 V for SCH 58261, and 125 V and 30 V for verapamil, respectively. Based on the full scan and MS/MS spectrum, the transitions of precursors to the product ions were as follows: *m*/*z* 346.1 → 105.1 for SCH 58261 and *m*/*z* 455.2 → 165.2 for Verapamil. Data acquisition and analysis were performed using the Analyst^®^ TF 1.6 software (Sciex, Foster City, CA, USA).

### 3.6. Method Qualification

The current LC–MS/MS assay was qualified in respect of linearity, intra-day and inter-day accuracy and precision and stability. The calibration curve was acquired by plotting the ratio between the peak area of SCH 58261 and the internal standard against the nominal concentration of calibration standards. The final concentrations of calibration standards obtained to plot the calibration curve were 3.02, 9.05, 27.16, 81.48, 244.44, 733.33, and 2200 ng/mL. The calibration curves were fitted by a weighted (1/concentration^2^) quadratic regression function, with an equation y = ax^2^ + bx + c. The criteria for the acceptability of the data were within ±25% accuracy and precision, except for the LLOQ, where it should not exceed ±30% accuracy, as well as precision. In addition, each run contained blank plasma samples with and without internal standards in duplicate.

Precision and accuracy were evaluated by determining the SCH 58261 concentrations in three replicates of QC samples freshly prepared in a daily base at three different concentrations for three separate days.

The dilution integrity experiment was carried out at five times the QC high concentration, i.e., 9100 ng/mL. Three replicate samples were prepared and their concentrations were calculated by applying the dilution factor of five against the freshly prepared calibration curve for SCH 58261. The acceptance criteria for dilution integrity were within ±25% precision and accuracy.

Stability assessments were performed to evaluate the SCH 58261 stability in stock solutions and plasma samples during study sample handling and analysis under different conditions. Stability assessments were performed by comparing the peak area ratio (SCH 58261 peak area/ISTD peak area) of the study samples against the freshly prepared control samples. The stability evaluations were conducted using triplicates of QC samples in four stability tests such as stock solution, short-term, long-term and freeze–thaw stability; stock solution stability after storage at −20 °C for 28 days; short-term stability after storage at room temperature for 4 h; long-term stability after storage at −80 °C for 28 days; freeze–thaw stability through three freeze–thaw cycles at −80 °C to 25 °C. Samples were considered to be stable if assay values were within ±25% accuracy and precision.

For species-dependent matrix effect tests, triplicate QC samples were prepared in mouse, dog, and human plasma. Samples were quantitated with a calibration curve prepared in rat plasma. The acceptance criterion was within ±25% of precision and accuracy.

### 3.7. Pharmacokinetic Study in Rats

All animal studies were performed in accordance with the “Guidelines in Use of Animal” established by the Chungnam National University Institutional Animal Care and Use Committee (Daejeon, Korea). This study was approved by the Chungnam National University Institutional Animal Care and Use Committee (No. CNU-01104). Male SD rats (8 weeks old; Samtako Biokorea, Gyeong-gi, Korea), 280–300 g, were used for the pharmacokinetic study. For the PK study, two groups of cannulated rats were administered a single 1 mg/kg intravenous dose and a single 5 mg/kg oral dose of SCH 58261. Blood samples were collected at 2, 5, 15, 30, 60, 90, 120, and 240 min following drug administration in the intravenous group (*n* = 3). Blood samples were collected 5, 30, 90 and 240 min for oral doses (*n* = 2). After centrifugation at 10,000 rpm for 5 min, plasma was transferred to Eppendorf tubes and stored at −20 °C until analysis. After sample preparation, the PK samples were analyzed by LC–MS/MS.

Pharmacokinetic parameters were obtained on each individual set of data using the Phoenix WinNonlin^™^ software (version 8.0; Pharsight Corporation, Mountain View, CA, USA) by non-compartmental analysis (NCA).

### 3.8. Parameter Sensitivity Analysis Using the Compartment Model

The compartment model of GastroPlus^™^ (version 9.5; Simulations Plus, Inc., Lancaster, CA, USA) was used for parameter sensitivity analysis. When simulating one-, two- and three-compartment models based on SCH 58261 structure, the most fitted model with the drug concentration that was observed after intravenous administration was selected. The selected model was a two-compartment model. The factors that influence the exposure of the drug after oral administration using the two-compartment model were examined through parameter sensitivity analysis. The drug exposure was assessed as AUC_last_. The total of seven factors were simulated for drug exposure: permeability, solubility, systemic clearance, renal clearance, FPE, blood–plasma ratio, and unbound fraction.

### 3.9. Microsomal Metabolic Stability

Rat liver microsomes (final concentration = 1 mg/mL) were incubated with SCH 58261 (1.5 µg/mL) in a reaction mixture that consisted of 2 mM NADPH and 5 mM UDPGA at 37 °C. All incubations were performed in triplicate and the reaction was initiated by adding the cofactor solutions containing NADPH and UDPGA to the rat liver microsome suspension with a 3-min pre-incubation. The microsomes, mixed-cofactor, and SCH 58261 were mixed and incubated for 0, 15, 30, and 60 min and the reaction was stopped by adding 50% ACN/50% methanol (MeOH) containing 100 ng/mL verapamil. After vortexing for 1 min and centrifuging at 8000× *g* for 10 min, the supernatant was transferred to another Eppendorf tube. Finally, the samples were diluted three times with DW and the mixture was transferred to an LC vial for LC–MS/MS analysis.

### 3.10. In Vitro Metabolite Identification

Rat liver microsomes (final concentration = 2 mg/mL) were incubated with SCH 58261 (5 µg/mL) in a reaction mixture that consisted of 2 mM NADPH, 5 mM UDPGA, and 0.5mM GSH at 37 °C. The reaction was initiated by adding the cofactor solutions containing NADPH, UDPGA, and GSH to the rat liver microsome suspension with a 3-min pre-incubation. The microsomes, mixed-cofactor, and SCH 58261 were mixed and incubated for 0 and 60 min at 37 °C. The reaction was stopped by adding ACN. After vortexing for 1 min and centrifuging at 8000× *g* for 10 min, the supernatant was transferred to another Eppendorf tube. The supernatants were evaporated to dryness under vacuum in a rotary evaporator with a cold trap (Eyela CVE-3110 & UT-1000, Tokyo, Japan). The dried residue was re-constituted to 210 μL of DW/MeOH (2:1), vortexed, centrifuged at 10,000× *g* for 5 min, and the supernatant was transferred to an LC vial for analysis. PeakView^®^ Version 2.2 (Sciex, Redwood City, CA, USA) and MetabolitePilot^™^ Version 2.0.2 (Sciex, Redwood City, CA, USA) were used for the structural elucidation of SCH 58261 metabolites.

### 3.11. Bile Duct-Cannulated Rat Study

A bile duct-cannulated rat study was conducted to evaluate the elimination pathway of SCH 58261. Samples of urine, bile, and feces were collected for 24 hours after a single 5 mg/kg oral dose (*n* = 1) from rat metabolic cages (JeungDo Bio & Plant co., Seoul, Korea). Urine samples were diluted twofold with 30% ACN. Feces samples were subsequently homogenized in four volumes of phosphate-buffered saline (PBS, fivefold dilution). The obtained samples were further processed and analyzed using LC–MS/MS.

## 4. Conclusions

SCH 58261 is one of the drugs being developed as an adenosine A**_2A_** receptor agonist and has been used in many papers as a reference drug in the development of an adenosine A**_2A_** receptor antagonist. However, there are no reports that clearly demonstrate the ADME/PK aspect of SCH 58261. Therefore, we investigated the ADME/PK of SCH 58261 as a reference drug for the development of adenosine receptor antagonists.

First, the LC–MS/MS method was established to accurately quantify SCH 58261 in rat plasma. The calibration curve range is 3.02 to 2200 ng/mL. This range was sufficient enough to cover the concentration of the drug when administered at 1 mg/kg intravenously and 5 mg/kg orally in rats. The correlation coefficient of the calibration curve showed a linearity of ≥0.99. The LC–MS/MS method has also been shown to be sensitive, selective, accurate, and reproducible through a variety of stability experiments (stock, short-term stability, long-term stability, and freeze–thaw stability) and achieved dilution integrity. Therefore, this method has been applied successfully to various in vitro and in vivo PK studies.

Then, in vivo PK studies for SCH 58261 were carried out in rats. The SCH 582631 was administered intravenously at 1 mg/kg and orally at 5 mg/kg, and the drug concentration in rat plasma was monitored. These studies showed that the bioavailability (BA) of SCH 58261 was extremely low when given orally and the root cause of the low BA was investigated.

A simple in silico method was used to find the root cause of the poor BA of SCH 58261 when given orally. The GastroPlus^™^ was used as an in silico tool. The parameter sensitivity analysis of GastroPlus^™^ examined the factors that influence the exposure of SCH 58261, such as AUC_last_. The factors that had a great effect on AUC_last_ for oral administration were permeability, systemic clearance, renal clearance, and liver FPE%. Therefore, we hypothesized that one (or a number) of these factors would have a significant effect on the low BA of SCH 58261.

Microsomal metabolic stability was performed to evaluate the metabolism of SCH 58261 in rat liver microsomes. The hepatic clearance value obtained from this experiment was 39.97 mL/min/kg. Assuming a rat liver blood flow of 55 mL/min/kg, the hepatic clearance value obtained was as high as 72% of the rat liver blood flow. Therefore, SCH 58261 is a drug that is highly metabolized by the liver, and it was supposed that one of the reasons for poor oral BA might be due to significant hepatic metabolism.

In vitro metabolite identification shows that the metabolite type of SCH 58261 in the rat liver is mono-oxidation or ketone-forming metabolites. A total of four metabolites were identified, three mono-oxidized metabolites and one metabolite-formed ketone. No significant metabolites greater than SCH 58261 appeared to be present, with an assumption of similar ionization efficiency between SCH 58261 and its metabolites.

Finally, a bile duct-cannulated rat study was conducted. Bile, urine, and feces samples from rats were collected to identify the main excretion route for SCH 58261 after oral administration. Approximately 28.17% of SCH 58261 administered was excreted in the first 24 h without absorption. This information implies that a major contribution to the extremely low BA of SCH 58261 would be likely due to no/little GI absorption after oral administration. Even if a small fraction of SCH 58261 was absorbed, SCH 58261 would be subject to significant drug metabolism by CYPs metabolic enzymes in the liver, which further contributed to the low BA of SCH 58261 in vivo. In conclusion, the low BA of SCH 58261 after oral administration in rats would be due to a limited absorption process (primary reason) with a high metabolism (secondary reason) in vivo.

In the future, researchers who are developing new SCH58261 analogs should be aware of the relationship between drug exposure and biomarkers in order to gain a better understanding of their pharmacokinetics/pharmacodynamics(PK/PD) for efficacy; otherwise, it would be challenging to predict efficacious doses in preclinical and clinical studies of the poor oral exposure of drugs. Several novel strategies to overcome this poor oral drug exposure, such as new formulations or pro-drug approaches, would warrant exploration for this purpose based on the PK and physicochemical properties of SCH58261 analogs.

## Figures and Tables

**Table 1:** Inter-/intra-day accuracy and precision of SCH 58261 in quality control samples (*n* = 3).

Run No.	Statistics	QC Low (15.04 ng/mL)	QC Medium (165.46 ng/mL)	QC High (1820 ng/mL)
1	Precision (% CV)	10.82	3.48	6.01
	Accuracy (%)	118.33	109.74	103.18
2	Precision (% CV)	1.89	1.21	5.84
	Accuracy (%)	124.09	107.54	97.33
3	Precision (% CV)	2.87	2.61	3.28
	Accuracy (%)	119.37	109.44	101.55
Inter-day	Precision (% CV)	5.20	2.43	5.04
	*n*	9	9	9
	Accuracy (%)	120.60	108.91	100.68

**Table 2:** The stability assessments of SCH 58261 in rat plasma (*n* = 3).

Assessments	Conditions	Statistics	QC Low (15.04 ng/mL)	QC Medium (165.46 ng/mL)	QC High (1820 ng/mL)
Stock stability	28 days −20 °C	Mean concentration	15.01	165.28	1821.58
		Accuracy (%)	99.82	99.89	100.09
		Precision (% CV)	12.23	2.56	5.55
Short-term stability	4 h 20 °C	Mean concentration	13.44	163.77	1836.01
		Accuracy (%)	89.38	98.98	100.88
		Precision (% CV)	8.53	8.24	5.32
Long-term stability	28 days −80 °C	Mean concentration	16.72	168.52	1694.76
		Accuracy (%)	111.14	101.85	93.12
		Precision (% CV)	6.58	3.30	3.13
Freeze-thaw stability	3 cycles −80 °C	Mean concentration	15.93	181.51	1893.10
		Accuracy (%)	105.92	109.70	104.02
		Precision (% CV)	10.69	4.80	2.12

**Table 3:** The dilution integrity assessments of SCH 58261 in rat plasma (*n* = 3).

Assessment	Dilution Factor	Statistics	QC Dil (9100 ng/mL)
Dilution integrity	5-fold	Mean concentration	7670.03
		Accuracy (%)	84.29
		Precision (% CV)	3.00

**Table 4:** Various species-dependent matrix effects in mouse, dog and human plasma (*n* = 3).

Assessment	Species	Statistics	QC Low (15.04 ng/mL)	QC Medium (165.46 ng/mL)	QC High (1820 ng/mL)
Species-dependent matrix effect	Mouse	Mean concentration	13.70	179.44	1718.69
		Accuracy (%)	91.11	108.45	94.43
		Precision (% CV)	11.02	10.13	12.50
	Dog	Mean concentration	13.93	155.11	1639.36
		Accuracy (%)	92.62	93.74	90.07
		Precision (% CV)	8.47	6.88	12.41
	Human	Mean concentration	15.21	169.72	1928.98
		Accuracy (%)	101.13	102.57	105.99
		Precision (% CV)	1.23	10.11	4.98

**Table 5:** Pharmacokinetic parameters of SCH 58261 after IV 1 mg/kg administration and PO 5 mg/kg administration in rats.

	Dose (mg/kg)	t_1/2_ (min)	T_max_ (min)	C_max_ (ng/mL)	AUC_last_ (min·ng/mL)	CL (mL/min/kg)	V_ss_ (mL/kg)	BA (%)
IV	1	52.21	3.00	1135.44	11,528.45	87.91	3196.92	0.03
PO	5	−	5.00	6.12	15.29	−	−	

**Table 6:** In vitro microsomal metabolic stability of SCH 58261 in rat liver microsomes.

Species	t_1/2_ (min)	CL_int, in vitro_ (mL/min/mg)	CL_int_ (mL/min/kg)	CL_H_ (mL/min/kg)
Rat	10.95	0.063	124.80	39.97

**Table 7:** Characteristics of in vitro SCH 58261 metabolite identification results by LC–MS/MS assay.

Peak ID	Name	Formula	Retention Time (min)	*m*/*z*	Nominal Mass Change (Da)
Parent	SCH 58261 [M + H]^+^	C18H15N7O	19.20	346.14	−
M1	Mono-oxidation	C18H15N7O2	13.61	362.14	+16
M2	Mono-oxidation	C18H15N7O2	14.13	362.14	+16
M3	Ketone formation	C18H13N7O2	15.25	360.12	+14
M4	Mono-oxidation	C18H15N7O2	15.76	362.14	+16

**Table 8:** Percentage of administered dose of SCH 58261 excreted in bile, urine, and feces following oral administration of 5 mg/kg in the rat.

Matrix	Time	Amount Excreted * (ng)	Excretion%
Bile	0–7 h	2094.89	0.14
	7–24 h	759.44	0.05
Urine	0–7 h	278.32	0.02
	7–24 h	81.06	0.005
Feces	0–24 h	422,516.74	28.17

* Drug amount excreted as SCH 58261.

**Table 9:** Mobile phase condition for LC gradients. (a) LC gradient for quantification, (b) LC gradient for metabolite identification.

	Time (Min)	Mobile Phase B (%)
(a)	0	10
	0.5	10
	0.9	95
	1.5	95
	1.6	10
	3.0	10
(b)	Time (Min)	Mobile Phase B (%)
	0	10
	1.0	10
	22.0	40
	24.0	95
	26.0	95
	26.1	10
